# Virus-Based MicroRNA Silencing and Overexpressing in Common Wheat (*Triticum aestivum* L.)

**DOI:** 10.3389/fpls.2017.00500

**Published:** 2017-04-10

**Authors:** Chao Jian, Ran Han, Qing Chi, Shijuan Wang, Meng Ma, Xiangli Liu, Huixian Zhao

**Affiliations:** ^1^College of Life Sciences, Northwest A&F University, YanglingChina; ^2^Crop Research Institute, Shandong Academy of Agricultural SciencesJinan, China; ^3^State Key Laboratory of Crop Stress Biology for Arid Areas, Northwest A&F UniversityYangling, China

**Keywords:** *Triticum aestivum* L., microRNA, artificial miRNA, silence, overexpression, BSMV

## Abstract

MicroRNAs (miRNAs) are a class of endogenous small non-coding RNAs that arise from large RNA precursors with a stem-loop structure and play important roles in plant development and responses to environmental stress. Although a hundred and nineteen wheat miRNAs have been identified and registered in the miRBase (Release 21.0, June, 2014; http://www.mirbase.org), the functional characterization of these miRNAs in wheat growth and development is lagging due to lack of effective techniques to investigate endogenous miRNA functions in wheat. Here we report barley stripe mosaic virus(BSMV)-based miRNA overexpression and silence systems that can be applied to study miRNA functions in wheat. By utilizing the BSMV system, we successfully knocked down endogenous miR156 and miR166 levels and over-expressed endogenous miR156 and artificial miRNA against phytoene desaturase gene *PDS* (amiR-PDS) in wheat. amiR-PDS expression caused a great reduction in endogenous mRNA abundance of *PDS* gene in wheat plant, leading to leaf obviously photobleaching. miR156 silencing led to a great increase in the mRNA level of its target gene *SPL2*, resulting in a leaf-curl phenotype in wheat seedlings. In contrast, overexpression of miR156 led to a significant reduction in the mRNA level of *SPL2* in wheat seedlings, resulting in serious delay of the vegetative phase transitions as well as booting and flowering in wheat. These confirmed that miR156 regulates wheat development and booting time through *SPL* genes. In summary, the BSMV-based miRNA overexpression and silence systems have extraordinary potential not only for functional study of protein-encoding genes but also for miRNA genes in wheat.

## Introduction

MicroRNAs (miRNAs) are a class of small endogenous 22 nucleotides (nt) to 24 nt RNAs that arise from large non-coding single-stranded RNA precursors, which are transcribed by RNA polymerase II to generate imperfect self-complementary, stem-loop secondary structures, and act as post-transcriptional regulators in eukaryotes (Bartel, 2004; Lee et al., 2004; Chen, 2009). In plant, mature miRNAs negatively regulate gene expression at the transcriptional or post-transcriptional levels through repressing gene translation or degrading targeted mRNAs (Chen, 2004; Voinnet, 2009). Therefore miRNAs play important roles in plant development (Ackerman and Sakai, 2003; Palatnik et al., 2003; Chen, 2009) and responses to biotic or abiotic stress (Sunkar et al., 2006; Xin et al., 2010). The first plant miRNA was identified in *Arabidopsis thaliana* (Llave et al., 2002). To date, approximately 8496 miRNAs have been identified in about 73 plant species, and all the information was deposited in miRBase (Release 21.0, June, 2014)^1^. However, the number of miRNA functionally characterized is very limited, and most of these miRNAs are from model plant species, such as Arabidopsis, rice (*Oryza sativa*) and tomato (*Solanum lycopersicum*) (Schommer et al., 2012; Zhang et al., 2013).

In Arabidopsis and rice, two reciprocal reverse genetic strategies have been used to identify the function of a particular miRNA. One is to highlight miRNA activity by transgenic overexpression of a miRNA in plant (Allen et al., 2007; Zhang et al., 2013). The other is to block miRNA function by identifying a mutant of a particular miRNA gene (Baker et al., 2005) or by expressing a miRNA-resistant target, which posses silent mutations being introduced to keep the encoded amino acids not be changed (Zhao et al., 2007), a miRNA target mimicry (MTM) (Franco-Zorrilla et al., 2007), or a short tandem target mimic (STTM) (Yan et al., 2012) in transgenic plant. However, all the techniques described above rely on time-consuming processes to produce stable transgenic plants, which limit their usage for high-throughput analysis in plant species with very low transformation efficiency. Allohexaploid common wheat (*Triticum aestivum* L., AABBDD; 2*n* = 6 × = 42), with very large complicate genome, is one of the major food crops worldwide. Therefore the improvement of wheat yield and quality has always been the most important target in wheat breeding programs. In fact, the yield potential of modern wheat varieties is fairly high, and further increasing wheat yield will largely depend on the knowledge of genes that control wheat growth and development (Cakir et al., 2010). Therefore, understanding the involvement of miRNAs in wheat development is crucial for wheat improvement, considering miRNAs as powerful endogenous regulators. It has been demonstrated that some miRNAs participate in many regulatory pathways that control seed development in Arabidopsis (*A. thaliana*) and rice (Nodine and Bartel, 2010; Zhang et al., 2013). For examples, miR156 targets *squamosa promoter-binding protein-like 10* (*SPL10*) and *SPL*11, and the regulation of these targets prevents premature gene expression during early embryogenesis in Arabidopsis (Nodine and Bartel, 2010). miR397 overexpression can improve rice seed size and promote panicle branching, increasing the grain yield up to 25% in field trial (Zhang et al., 2013). To date 119 miRNAs have been registered in the miRBase/*T. aestivum* (Release 21.0, June 2014)^2^, however, the function of these miRNAs in wheat growth and development is still unknown. Actually, none of all the aforementioned techniques used for miRNA functional identification can be applied to wheat, due to the large complicate genome and very low transformation efficiency which pose a technical challenge to produce numbers of transformants needed to saturate the wheat genome.

The approaches of virus-induced transient gene silencing (VIGS) and gene expressing in plants have been developed as an effective genetics tool for assessing gene functions (Dalmay et al., 2000; Lu et al., 2003; Scofield et al., 2005; Tang et al., 2010; Ma et al., 2012; Sha et al., 2014). Cabbage leaf curl virus (CaLCuV) can infect a range of dicotyledons, including cabbage (*Brassica capitata*), tobacco (*Nicotiana benthamiana)*, and Arabidopsis (Hill et al., 1998), and a recombinant CaLCuV-based vector was first developed to trigger siRNA-mediated silencing in Arabidopsis (Turnage et al., 2002). Furthermore, the CaLCuV vector was modified to express artificial and endogenous miRNAs in tobacco (Tang et al., 2010). In addition, tobacco rattle virus (TRV) can also infect a broad range of dicot plant species. TRV-based vectors were developed and widely used as VIGS vectors to knock down gene expression in various plant species (Liu et al., 2002; Bachan and Dinesh-Kumar, 2012), and they were also modified for expressing foreign genes in plants (MacFarlane and Popovich, 2000). More recently, the TRV vector was modified into a TRV-based T-DNA expression vector, and a virus-based microRNA silencing system, in which endogenous miRNA activity can be effectively suppressed by TRV-based expression of miRNA target mimics, was developed to silence endogenous miRNAs in plant (Sha et al., 2014). Barley stripe mosaic virus (BSMV) is a positive sense, single-strand RNA hordeivirus with a tripartite genome, i.e., α, β, and γ RNAs (Petty et al., 1989). Because the BSMV can infect monocotyledon barley and wheat plants, BSMV-based vector has been developed and used for gene silencing in wheat seedlings (Scofield et al., 2005). A protocol of BSMV-induced gene silencing in wheat spike/grain has also been established and used to identify functional genes involved in seed development of wheat by our group (Ma et al., 2012, 2015, 2016). More recently, the BSMV vector was successfully used to silence endogenous miRNAs in wheat by expressing miRNA target mimics (Jiao et al., 2015). However, there is no report on approaches of miRNA expressing in wheat and barely.

Our long-term goal is to reveal the functions of endogenous miRNAs in wheat development. The purpose of the present study is to develop approaches for applying the BSMV-based vector to efficiently block endogenous miRNA activity by expressing STTMs of a particular miRNA and to express a particular endogenous miRNA or artificial miRNA (amiRNA) by expressing the pre-miRNA/pre-amiRNA in wheat.

## Materials and Methods

### Plant Material and Growth

Spring wheat cultivar Ningchun 16, which was previously demonstrated to be infectivity by the BSMV (Ma et al., 2012), was used in this experiment. Wheat seeds were germinated in a controlled growth chamber (25–27°C), and then wheat plants were grown in a green house with a light period of 16 h/day (regulated with supplementary light), a day/night temperature regime of 20–25°C/15–18°C, and 65–75% relative humidity, and watered as needed. The wheat plants were exposed to a temperature of 4°C for 14 days to achieve complete vernalization at the two-leaf stage.

### Construction of BSMV-Derived Vectors

**FIGURE 1:**
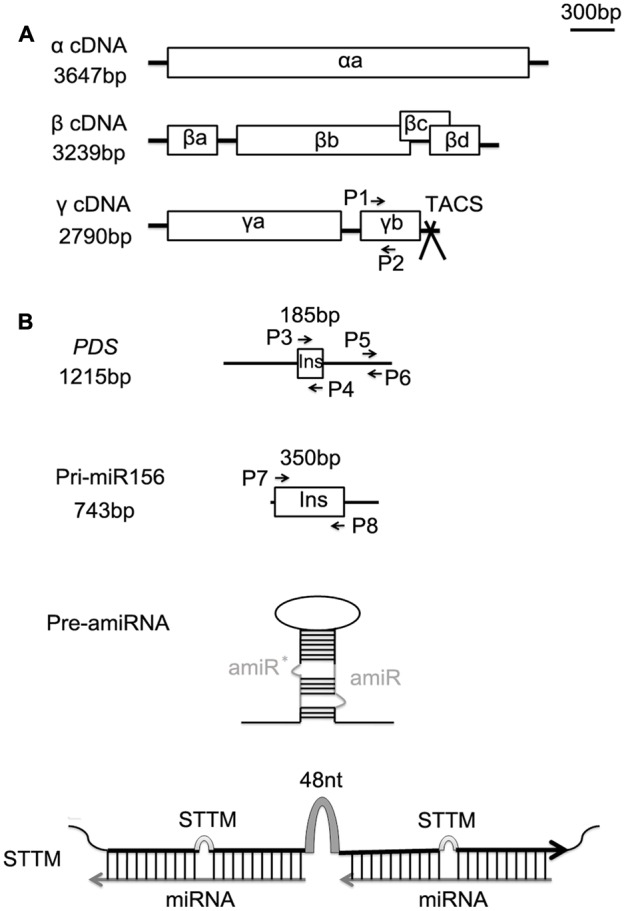
**Schematic organization of the *Barley stripe mosaic virus* (BSMV) genomes and the inserts used for BSMV-induced miRNA silencing or overexpressing. (A)** Genomic organization of the three BSMV components α, β, and γ drawn to scale of 300 bp. Open reading frames are indicated by boxes. The TA cloning site (TACS) designed for direct cloning of PCR products is positioned after the stop codon of the γb gene. **(B)** Schematic representation of full-length phytoene desaturase gene (*PDS*) and primary sequence of tae-miR156 (pri-miR156) (black line) drawn to the same scale as above (the upper two panes), and the structures of an artificial miRNA precursor (pre-amiRNA) and a STTM module (the lower two panes). The 185-bp fragment of *PDS* and the 350-bp fragment of pri-miR156 indicated by boxes, and the sequences of pre-amiRNA and STTM are inserted into BSMV γ vector for virus induced the target fragment expressing or silencing. P1 and P2 are primers for semiquantitative RT-PCR analysis of γ*b*. P3 and P4, P7 and P8 are primer sets for generating the virus vectors BSMV:PDS and BSMV:miR156, respectively. P5 and P6 are primer pair for quantitative real-time RT-PCR (qRT-PCR) analysis of endogenous *PDS* in wheat. Pre-amiRNA contains an amiRNA against *PDS* gene along with miR319a precursor of Arabidopsis (EU549293) as backbone, ^∗^indicates star sequence of a miRNA. STTM contains two tandem target mimics separated by a 48-nucleotide imperfect stem-loop linker (48 nt).

The BSMV vectors, which contain α, β, and γ constructs, used in this experiment were kindly provided by Dr. Huang Li at Montana State University, Bozeman, USA. The schematic organization of the BSMV genomes was shown in **Figure 1A**. The γ construct was modified to include a polymerase chain reaction (PCR)-ready cloning site (TA-cloning site, TACS) following the protocol described previously (Ma et al., 2012). The BSMV γ RNA construct carrying a 185-bp fragment of the barley (*Hordeum vulgare* L.) phytoene desaturase gene (*PDS*) with the gene in antisense orientation was also provided by Dr. Huang Li (**Figure 1B**). The recombinant BSMV vector containing the *PDS* fragment (named as BSMV:PDS4as) can efficiently induce *PDS* silencing in wheat leaves (Scofield et al., 2005). We utilized the BSMV:PDS4as as a positive control for testing virus-induced expression of an amiRNA against the mRNA of wheat *PDS* gene (amiR-PDS). For simplicity, BSMV:PDS was used to stand for the BSMV: PDS4as vector, and BSMV:00 for the BSMV with no insert (empty vector) in our experiments.

The BSMV vector carrying a fragment of precursor sequences of wheat miR156 (pre-miR156, 350 bp in length) (**Figure 1B**), designed as BSMV:miR156, was constructed for miR156 overexpressing in wheat plants. The wheat pre-miR156 sequence available on miRBase^3^ was predicted based on the sequence of primary transcript of miR156 (pri-miR156) (Genbank accession CL902915) in wheat (Dryanova et al., 2008). A primer pair P7/P8 was designed according to the sequence of the pri-miR156 for amplifying the pre-miR156 sequences by PCR, with the genome DNA of Ningchun 16 as a template. The resulting PCR products were purified and directly inserted into the BSMV γ construct by T-A cloning in either the sense (BSMV: miR156-F) or antisense (BSMV: miR156-R) orientation.

The BSMV vector containing an amiR-PDS precursor, named as BSMV:amiR-PDS, was generated for amiRNA expression in wheat. To obtain the BSMV:amiR-PDS, a Web-based tool Web MicroRNA Designer^4^ was used to design a 21-mer amiR-PDS sequence, and the resulting sequence is 5′-UAAUCUGUUUAGAGGAAUCAG-3′. To generate the amiR-PDS precursor sequence, we conducted PCR amplification according to the strategies previously described (Tang et al., 2010; Gu et al., 2014). In brief, four specific oligonucleotide sequences (amiR-PDS-I, amiR-PDS-II, amiR-PDS-III, and ami-PDS-IV) suggested by Web MicroRNA Designer were used to amplify the designed amiR-PDS along with primer pair P9/P10, using the endogenous miR319a precusor of Arabidopsis (EU549293) as backbone. While, the full-length miR319a precursor was amplified from Arabidopsis. The detailed protocol for generating amiR-PDS precursor can be found in Supplementary Method S1. The resulting amiR-PDS precursor was inserted into BSMV γ construct by T-A cloning.

The BSMV vectors utilized to silence endogenous particular miRNAs were constructed to carry STTM of the certain miRNA, which is composed of two short sequences mimicking small RNA target sites separated by a linker of an 48-nucleotide RNA spacer (totally 153 bp in length) (**Figure 1B**), and designed as BSMV:STTM, including BSMV:STTM156/156 and BSMV:STTM166/166 which harbors two copies of short tandem target mimic of miR156 and miR166, respectively (**Figures 3A, 4A**). The STTM156/156 and STTM166/166 sequences were designed abiding by the rules for STTM designing reported previously (Yan et al., 2012), and synthesized by Soagon Biotech (Shanghai). Each of these two STTM modules was separately inserted into TACS of the BSMV γ construct. This was done by PCR amplification of the synthesized STTM sequences with a pair of universal PCR primer (the forward primer P11: 5′-GTTGTGTGGAATGTATGGAGC-3′ and the reverse primer P12: 5′-GCTGTAATCACACTGGCTCA-3′) complementary to the 5′- and 3′-end of the sequences of the STTM modules designed in advance, and the resulting PCR products were purified and directly inserted by T-A cloning. The sequences of all the primer pairs used in this study were listed in Supplementary Table S1.

### *In vitro* Transcription of Viral RNAs and Plant Inoculations

*In vitro* transcription of viral RNAs was conducted according to the procedure previously described (Scofield et al., 2005). The BSMV RNAs *in vitro* synthesized were rub-inoculated onto the 2nd or the 3rd fully expanded leaf of wheat at 3- or 4-leaf stage. Ten plants were infected with each of the BSMV:amiR-PDS, the BSMV:miR156, the BSMV:STTM156/156, the BSMV:STTM166/166, the BSMV:PDS, and the BSMV:00, respectively, as described previously (Scofield et al., 2005; Ma et al., 2012), the inoculation with BSMV:PDS and BSMV:00 being as a positive control, and a control, respectively. Three independent biological replicates were included in this experiment.

### RNA Isolation and cDNA Synthesis

For analyzing the abundance of a particular mature miRNA and its target mRNA in wheat infected with each reconstructed BSMV, the 2nd leaves upper the inoculated ones were collected at 2, 4, 8, 12, 15, 20, 22, and 30 days post inoculation (dpi), with three biological replicates included. Total RNA was extracted from each of these leaf samples by using TRIzol reagent (Invitrogen, Grand Island, NY, USA) according to the instructions the manufacturer recommended, and treated with RNase–free DNase I (TaKaRa, Dalian, China). The integrity of each RNA sample was checked by electrophoresis of 1% agarose gel, and the concentration was determined with a Nanodrop ND-1000 spectrophotometer (Nano Drop Technologies, Wilmington, DE, USA). For detection of coding genes, first-strand cDNA was synthesized using 1 mg of total RNA with oligo (dT) primer and MMLV reverse transcriptase (TaKaRa, Dalian, China), and for detecting STTM mudules, first-strand cDNA was synthesized using the primer P12 specific to STTM. For testing mature miRNA, the specifically designed stem-loop reverse transcription primer for each of miRNA was used to reverse-transcribe (RT) individual miRNA, primer P13 and P14 being used for RT-PCR of miR156 and miR166, respectively, with 1 mg of total RNA, following the procedures described previously (Chen et al., 2005; Han et al., 2014). The resulting cDNA was diluted and used for further study.

### RT-PCR and Quantitative Real-Time RT-PCR

To detect the abundance of the BSMV RNA and the inserts, including pre-miR156, pre-amiR-PDS, and STTM module, in the infected plants, the cDNAs from the leaf samples collected at 12 dpi were used for semiquantitative RT-PCR analysis, primer pair P1/P2 being used for detecting the γ RNA, P7/P8 for pre-miR156, P9/P10 for pre-amiR-PDS, and P11/P12 for STTM 156/156. The primer pair P15/P16 for an internal conference gene *Glyceraldehyde-3-phosphate dehydrogenase (GAPDH)*, which is constitutively expressed in wheat, was used for cDNA normalization (Sun et al., 2010). All the sequences of the primer pairs used were listed in Supplementary Table S1.

To determine the abundance of the interested mature miRNAs and their target mRNAs in wheat infected with BSMV:STTM156/156, BSMV:STTM166/166, BSMV:amiR-PDS, and BSMV:miR156, respectively, quantitative real-time RT-PCR (qTR-PCR) was performed with *U6* (small U6 spliceosomal RNA) and *GADPH* as internal reference genes for cDNA normalization. Forward primer P17 and P18 specific for miR156 and miR166, respectively, were separately used with universal reverse primer P19 to detect mature miR156 or miR166 levels, and primer pair P20/P21 for U6 was used for cDNA normalization. Primer pair P22/P23 was used for miR156 target sequence (GenBank ID: CK196549)^5^, which is the homolog of *SPL2* in *O. sativa* and *Brachypodium distachyon*, and P24/P25 was used for *Homeobox-leucine zipper protein HOX33-like* (Accession no.*Ta#S52543234*), which was predicted to be one of the miR166 target in wheat (Sun et al., 2014). The primer pair P15/P16 for *GAPDH* was used for cDNA normalization of encoding genes. Real-time RT-PCR with three technical replicates were performed on a CFX96 real-time system (BIO-RAD, USA) by using SYBR Premix Ex TaqTM II (TaKaRa, Dalian, China). The relative abundance of each miRNA and its target mRNA was calculated by the 2^-ΔΔCT^ method as previously reported (Livak and Schmittgen, 2001). All primers used in this experiment were listed in Supplementary Table S1.

## Results

### A BSMV-based Vector Is Able to Overexpress Its Inserts in Wheat

**FIGURE 2:**
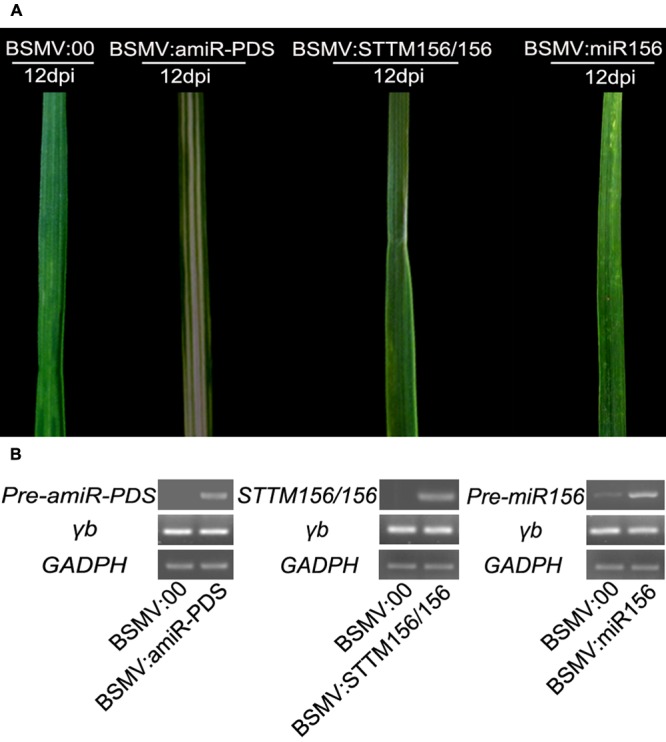
**A BSMV-based vector that can express its insert fragments in wheat.** Wheat cv. Ningchun 16 was infected with *in vitro* transcribed RNAs representing the α, β, and γ of BSMV:00; α, β, and γ-PDS RNAs of BSMV:PDS; α, β, and γ-STTM156/156 of BSMV:STTM156/156 and α, β, and γ-pre-miR156 of BSMV:miR156 onto the 2nd leaves of seedlings. **(A)** The phenotypes of the 4th leaves from wheat plants infected with BSMV:00, BSMV:amiR-PDS, BSMV:STTM156/156 and BSMV:miR156 at 12 days post inoculation(dpi), respectively. **(B)** Semiquantitative RT-PCR assays detect the over-expression of the pre-amiR-PDS, STTM156/156, and pre-miR156 in the leaves shown in **(A)**.

To investigate whether a BSMV-based vector can be used to block miRNA activity or to overexpress a mature miRNA, we first tested the possibility of the BSMV-derived vector causing accumulation of the inserts it carried in wheat. We inoculated the 2nd fully expanded leaves of wheat at three-leaf stage with BSMV:00, BSMV:amiR-PDS, BSMV:STTM156/156, and BSMV:miR156, respectively. Different phenotypes were observed in the 4th leaves of the infected plants at 12 dpi (**Figure 2A**). The leaves of the plants infected with BSMV:amiR-PDS exhibited photobleaching phenotype, those infected with BSMV:STTM156/156 displayed shrinking in the middle section of the leaves, while those infected with BSMV:miR156 did not showed any obviously symptom, presenting mosaic and chlorotic stripes similar as that of the plants infected with BSMV:00. Semiquantitative RT-PCR analysis was conducted to detected the abundance of the BSMV γ RNA, pre-amiR-PDS, STTM156/156, and pre-miR156 in the leaves exhibited in **Figure 2A**. The results showed that the BMSV γ RNA and the inserts were accumulated in the leaves of the infected plants at 12 dpi (**Figure 2B**), suggesting that a BSMV-based vector is able to overexpress the inserts it carried in wheat.

### miR156 and miR166 Can Be Silenced in Wheat by Using BSMV Vectors

**FIGURE 3:**
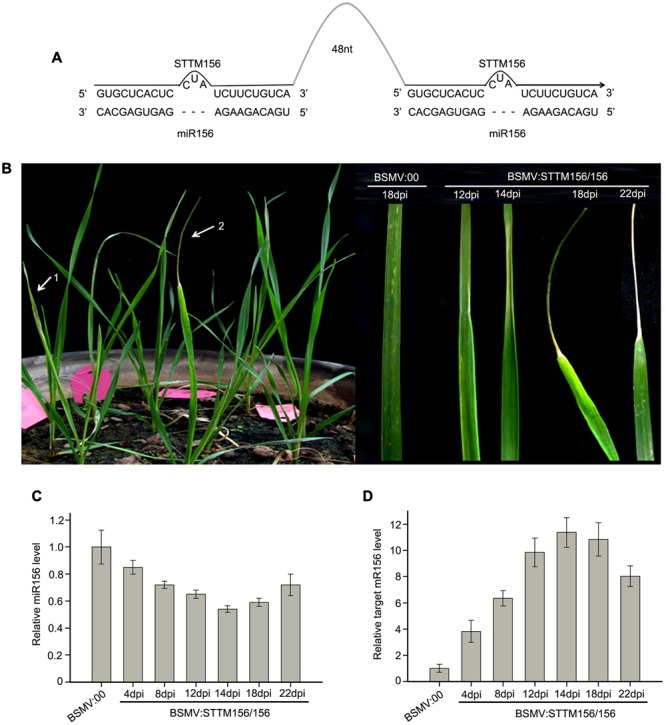
***Barley stripe mosaic virus*-derived vector BSMV:STTM156/156 can induce endogenous miR156 silencing in wheat. (A)** Diagram of STTM156/156. 48 nt, 48-nucleotid imperfect stem-loop linker. **(B)** The phenotype of leaves from wheat infected with BSMV-based vectors. 1 and 2 in left pane showed the 4th leaves of wheat plants infected with BSMV:00 and BSMV:STTM156/156 at 18 days post inoculation (dpi), respectively. The right pane is the 4th leaves of the infected wheat plants photographed at 12, 14, 18, and 22 dpi, respectively. The leaves shown are representative of differently treated plants. **(C)** The relative abundances of miR156 in the leaves of wheat inoculated with BSMV:STTM156/156 at 4, 8, 12, 14, 18, and 22 dpi are determined by Stem-loop RT-PCR (with U6 as a reference gene to normalization), compared to that of the control plants inoculated with BSMV:00. Each column represents the mean of three samples, and error bars indicate the standard deviation. **(D)** The mRNA levels of miR156 target *SPL2* (CK196549) in the leaves shown in **(C)** determined by quantitative real time RT-PCR. Error bars representing the standard deviation were calculated from three replicates.

**FIGURE 4:**
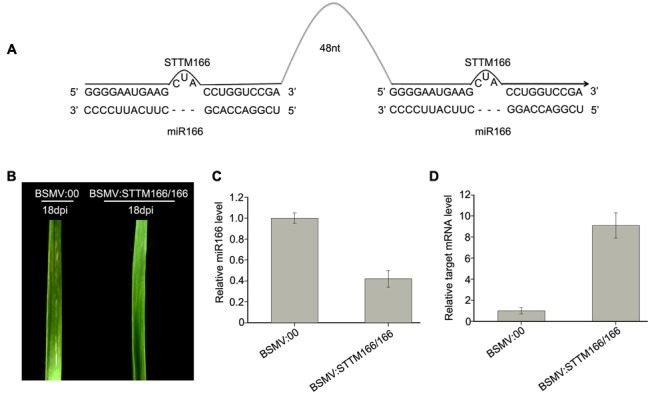
***Barley stripe mosaic virus*-derived vector BSMV:STTM166/166 induces endogenous miR166 silencing in wheat plants. (A)** Diagram of STTM166/166. 48 nt, 48-nucleotid imperfect stem-loop linker. **(B)** The 4th leaves of wheat plants infected with BSMV:00 and BSMV:STTM166, respectively, photographed at 18 days post inoculation (dpi). The leaves shown are representatives of 10 plants treated differently. **(C)** The miR166 levels in the leaves shown in **(B)** detected by Stem-loop RT-PCR. Each column represents the mean of three samples, and error bars indicate the standard deviation. **(D)** The mRNA levels of miR166 target gene *homeobox-leucine zipper protein HOX33-like* (*Ta#S52543234*) in the leaves shown in **(B)** determined by real-time RT-PCR. Error bars representing the standard deviation were calculated from three replicates.

miR156 and miR166 are conserved among plant species and highly expressed throughout the growing period of wheat (Han et al., 2014). It has been demonstrated that the miR165/166 family target and repress the expression of target gene *homeodomain-leucine zipper* (*HD-Zip*) *III*, determining the behavior of apical dominance and the phenotype of ectopic leaf growth and development in Arabidopsis (Jung and Park, 2007; Yan et al., 2012) and in *N. benthamiana* (Sha et al., 2014). miR156 targets the SQUAMOSA PROMOTER BINDING PROTEIN-LIKE (SPL) transcription factor genes which are involved in the promotion of vegetative phase transitions as well as flowering (Wu and Poethig, 2006). It has been demonstrated that the functional blockage of miR156/157 triggered an early vegetative phase change and early flowering in *A. thaliana* (Yan et al., 2012). Therefore, we chose miR156 and miR166 as target miRNA to knock down in wheat in order easy to check whether a BSMV-derived vector can induce endogenous miRNA silencing in wheat or not. So, we constructed BSMV:STTM156/156 and BSMV:STTM166/166 (**Figures 3A, 4A**), and inoculated the wheat plants with these BSMV-derived vectors. First, we inoculated wheat plants onto the 2nd fully expanded leaves with BSMV:STTM156/156 and BSMV:00, respectively. The experiment included three independent biological replicates and each replicate contains ten wheat plants. As previously observed, the symptoms of wheat infected with BSMV:STTM156/156 were indistinguishable from those infected with BSMV:00 at 10 dpi or earlier. However, the wheat plants inoculated with BSMV:STTM156/156 appeared to shrink in the middle section of the 4th leaves at 12 dpi, apparently curled at 14 dpi, and further curled up into a needle-like leaves at 18 dpi, but the leaves remained green, while the needle-like part of the leaves began to wither 22 dpi later (**Figure 3B**). This phenotype was rarely found in the 5th leaves of these plants. No leaf-curling phenomenon was observed in the wheat infected with BSMV:00. To confirm that the observed leaf-curling phenomena were the result of miR156 silencing, the 4th leaves of the BSMV:STTM156/156 infected plants were collected at 4, 8, 12, 14, 18, and 22 dpi to measure the abundance of endogenous mature miR156 by qRT-PCR described in section “Materials and Methods.” The results indicated that the miR156 levels were significantly knocked down in BSMV:STTM156/156 infected plants from 4 to 22 dpi (**Figure 3C**). About 15% reduction in mature miR156 abundance was detected as early as 4 dpi, and a remarkable reduction of miR156 level was detected at 14 ∼ 18 dpi. The needle-like leaves were seen at 18 dpi, much later than the reduction of the miR156 level. It is known that the mRNA sequence (GenBank ID: CK196549) is a homolog of miR156 target gene *SPL2* in *B. distachyon* by sequence alignment, therefore, we expect that the CK196549 is one of the miR156 target genes in wheat. Thus, we conducted qRT-PCR to analyze the level of the CK196549 in the leaves of the infected wheat. Indeed, the levels of the CK196549 were significantly higher in BSMV:STTM156/156 infected plants than in control plants from 4 to 22 dpi (**Figure 3D**). Contrasting to the reduction of miR156 abundance, a remarkable increasing of mRNA abundance of the miR156 target gene was detected at 14 dpi. These suggested that BSMV:STTM156/156 can induce miR156 silencing in wheat plants.

Then, we inoculated wheat plants with BSMV:STTM166/166 and BSMV:00, respectively, with three replicates included and each replicate contains 10 wheat plants, in order to further confirm the conclusion that a BSMV-based vector can induce miRNA silencing in wheat plants. Similarly, the wheat infected with BSMV:STTM166/166 and BSMV:00 did not show any distinguishable symptoms at 10 dpi or earlier. However, the plants infected with BSMV:STTM166/166 showed a slightly different phenotype from those control plants in the 4th leaves from 12 to 30 dpi. The BSMV:STTM166/166 infected plants exhibited a symptom of leaf-twist in the 4th leaves, while the control plants did not (**Figure 4B**). Our observation mirrored the damage of leaf primordial functions generated by the inhibition of miR166. This result is consistent with the previous report (Yan et al., 2012). *Homeobox-leucine zipper protein HOX33-like* (*Ta#S52543234*) was predicted to be one of the miR166 target in wheat (Sun et al., 2014). Analysis of qRT-PCR confirmed that the miR166 abundance was significantly knocked down in the BSMV:STTM166/166 infected plants (**Figure 4C**). In contrast, the mRNA level of *Ta#S52543234* in the BSMV:STTM166/166 infected plants was much higher than that in control plants (**Figure 4D**).

Taken together, the above results suggested that the BSMV vectors carrying STTM sequences can effectively block endogenous miRNA activity in wheat.

### Overexpress miR156 in Wheat Using a BSMV-Derived Vector

**FIGURE 5:**
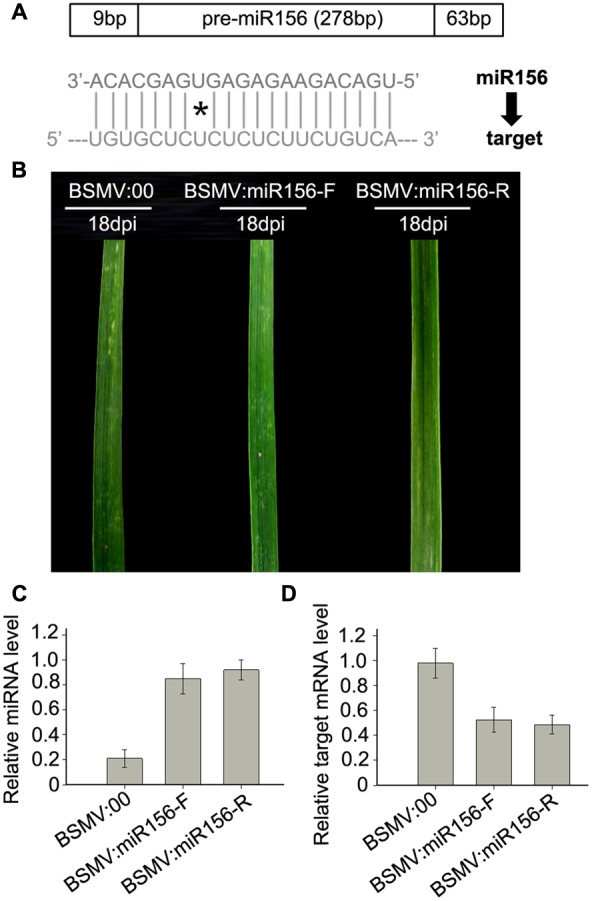
**miR156 overexpression induced by a BSMV-based vector leads to obvious reduction of mRNA level of miR156 target gene *SPL2* in wheat. (A)** Diagram of pre-miR156-containing fragment (the upper) and mature miR156 against its target (the lower), ^∗^presents mismatched base pair. **(B)** The 5th leaves of wheat plants infected with BSMV:00, BSMV:miR156-F and BSMV:miR156-R, respectively, photographed at 18 days post inoculation (dpi). **(C)** The mature miR156 levels in the leaves shown in **(B)** determined by Stem-loop RT-PCR. Each column represents the mean of three samples, and error bars indicate the standard deviation. **(D)** The mRNA levels of miR156 target *SPL2* (CK196549) in the leaves shown in **(B)** detected by real-time RT-PCR. Error bars representing the standard deviation were calculated from three replicates.

**FIGURE 6:**
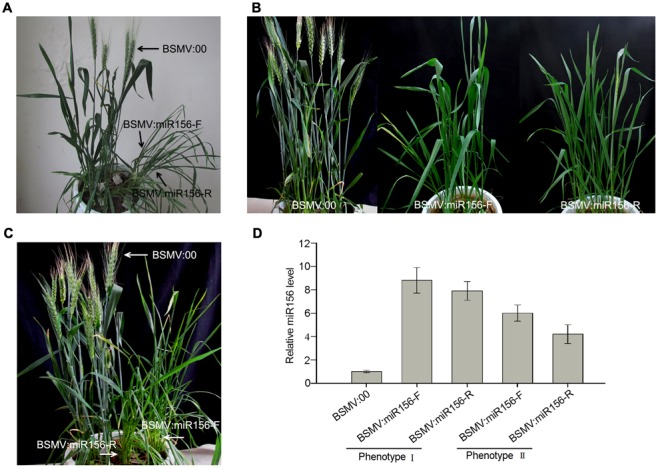
**Two phenotypes of wheat infected with BSMV:miR156 constructs. (A,B)**, Phenotype I and II of wheat inoculated with BSMV-derived vectors BSMV:miR156-F and BSMV:miR156-R, respectively, photographed at 28 days post inoculation (dpi). **(C)** Phenotype I of wheat inoculated with BSMV-derived vectors photographed at 40 dpi. **(D)** The mature miR156 levels in the wheat plants shown in **(A,B)** detected by Stem-loop RT-PCR. Each column represents the mean of three samples, and error bars indicate the standard deviation.

To test whether a BSMV-derived vector can be used to over-express endogenous miRNA to silence target genes in wheat, we constructed BSMV-derived vector BSMV:miR156-F and BSMV:miR156-R, which carries pre-miR156 sequences (upper pane in **Figure 5A**) in the sense and antisense orientation, respectively. Ten wheat seedlings gone through vernalization were inoculated onto the 3rd fully expanded leaves with BSMV:miR156-F, BSMV:miR156-R, and BSMV:00, respectively, at 4-leaf stage. It was observed that the wheat infected with BSMV:miR156-F, BSMV:miR156-R or BSMV:00 did not show any distinguishable symptoms at 18 dpi or earlier (**Figure 5B**). However, qRT-PCR analysis revealed that the abundances of miR156 were greatly increased in the leaves of wheat infected with BSMV:miR156-F or BSMV:miR156-R, compared to that in control plants (**Figure 5C**), on the contrary, the levels of miR156 target CK196549 were largely reduced in the leaves of plants infected with BSMV:miR156-F or BSMV:miR156-R (**Figure 5D**). Further observation found that the wheat plants infected with BSMV:miR156-F and BSMV:miR156-R showed very different phenotypes from control plants at 20 dpi and later. The control plants exhibited normally booting, heading and flowering (**Figures 6A–C**), however, the wheat plants infected with either BSMV:miR156-F or BSMV:miR156-R displayed two different phenotypes: type I (plants) showed the increased tiller number (5 more than the control plants) but not booting and heading (**Figures 6A,C**), and type II (plants) exhibited the similar tiller number as control plants did but with very late booting and heading date (7–10 days later than the control plants) (**Figure 6B**), the control plants heading at 25 dpi whereas the BSMV:miR156-infected wheat heading at about 32 dpi. These observation reflected the delay of the vegetative phase transitions and heading as well as flowering caused by miR156 overexpression resulting in great reduction of the levels of the target mRNA CK196549 (Wu and Poethig, 2006). We repeated this experiment five times, with ten plants for each construct in each experiment, and similar results were obtained. Furthermore, qRT-PCR analysis of miR 156 abundance was conducted to investigate if the different phenotype caused by distinct mature miR156 abundances leading to different mRNA levels of the miR156 target in these BSMV-miR156-infected wheat. The results confirmed that the mature miR156 levels in type I plants were significantly higher than in type II plants (**Figure 6D**). Taken together, a BSMV-based vector is able to overexpress endogenous miRNAs to silence target genes for rapidly studying miRNA functions in wheat.

### A BSMV-Based Vector Is Able to Express amiRNAs to Silence Target Genes in Wheat

**FIGURE 7:**
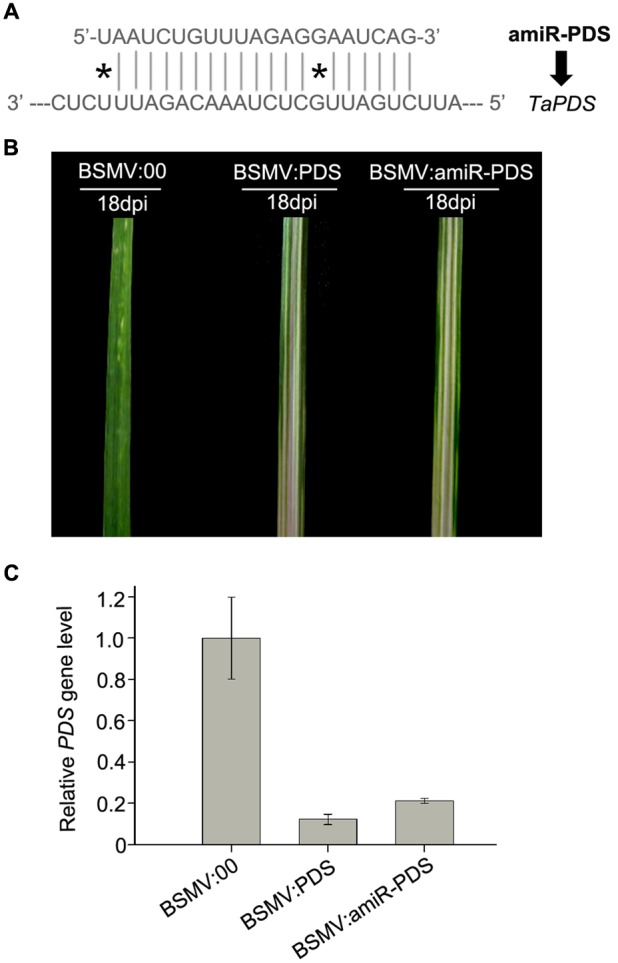
**Expression of amiR-PDS induced by BSMV-based vector caused *PDS* gene silencing in wheat. (A)** The sequences of artificial miRNA (amiR-PDS) against the mRNA of its target *TaPDS*, ^∗^ presents mismatched base pair. **(B)** The 5th leaves of wheat plants infected with BSMV:00, BSMV:amiR-PDS and BSMV:PDS, respectively, photographed at 18 days post inoculation (dpi). **(C)** The mRNA levels of *PDS* in the leaves shown in **(B)** detected by Stem-loop RT-PCR. Each column represents the mean of three samples, and error bars indicate the standard deviation.

In recent years, amiRNA technique has been established by using an endogenous miRNA precursor to yield a new target-specific miRNA for gene silencing in plants (Schwab et al., 2006; Molnar et al., 2009). And amiRNA has been demonstrated to specifically and efficiently silence single or multiple target genes by a well-designed precursor sequence (Schwab et al., 2006). To examine whether a BSMV-based vector is able to express amiRNAs to silence endogenous target genes in wheat, we designed an amiRNA targeting *PDS* gene in wheat and inserted it into the BSMV vector to obtain BSMV:amiR-PDS (**Figures 1B, 7A**). We inoculated ten wheat plants onto the 3rd fully expanded leaves with BSMV:amiR-PDS (for silencing *PDS*), BSMV:PDS (for positive control) and BSMV: 00 (control), respectively. As expected, mosaic and chlorotic stripes were observed on the tip portions of the 4th leaves of all inoculated plants at 7 dpi. Photobleaching was observed only at the 5th leaves of the wheat infected with BSMV:amiR-PDS or BSMV:PDS during 10 ∼ 30 dpi (**Figure 7B**). qRT-PCR analysis further confirmed that the endogenous *PDS* level, similar as that in the BSMV:PDS infected plants, was largely decreased in BSMV:amiR-PDS infected plants (**Figure 7C**). These suggested that the mRNA abundance of endogenous *PDS* in the BSMV:amiR-PDS infected plants was knocked down by amiR-PDS expression, resulting in leaf photobleaching in these plants. Thus, the BSMV-derived vector is capable of expressing target-specific artificial miRNAs to silence endogenous genes in wheat.

## Discussion

In the present study, we demonstrated that a BSMV-derived vector can be used to silence endogenous target miRNAs and overexpress amiRNAs or endogenous miRNAs in wheat. By utilizing the BSMV systems, we successfully knocked down the levels of endogenous miR156 and miR166 and over-expressed endogenous miR156 and amiR-PDS in wheat. miR156 silencing resulted in a great increase in the abundance of its target mRNA, and led to a leaf-curl phenotype in wheat seedlings (**Figure 3**), suggesting that miR156 plays important roles in leaf development by regulating its target gene expression in wheat seedlings. While miR166 silencing led to a large increase in the mRNA level of its target gene *Homeobox-leucine zipper protein HOX33-like*, and generated a leaf-twist phenotype in wheat seedlings (**Figure 4**), indicating that miR166 functions in wheat growth and leaf status. Similar results were obtained by CaLCuV-induced amiR156 expressing in *N. benthamiana* (Tang et al., 2010) and by TRV-mediated miR165/166 siliencing in *N. benthamiana* and Arabidopsis (Sha et al., 2014). In Arabidopsis, 11 of 17 SPL transcription factor genes have miR156 binding-site, and miR156 targets the *SPL* genes, including *SPL3, SPL4*, and *SPL5*, which are involved in the promotion of vegetative phase transitions as well as flowering (Wu and Poethig, 2006). miR156 has also been reported to function in rice development (Xie et al., 2006). More recently, 58 *SPL* genes were identified in wheat genome, and functional characterization of two *SPL*s, *TaSPL3* (with miR156 site mutated) and *TaSPL6*, found that they involved in regulation of flowering time and biomass accumulation (Wang et al., 2015). The diverse gene structures and different expression patterns suggested that *SPL* genes have a wide range of functions in wheat (Zhang et al., 2014). In this study, overexpression of endogenous miR156 in wheat seedlings led to a significant reduction in the levels of the miR156 target mRNA (**Figure 5**), resulting in distinct phenotypes of either not booting or booting and heading very late (**Figure 6**). This suggested that miR156 overexpression delays or prevents booting and flowering by inhibiting the expression of its target mRNA (CK196549) in wheat, which depends on the degree of reduction in the level of the mRNA in wheat. The present study confirms that miR156 regulates the vegetative phase transitions and booting as well as flowering through *SPL* genes in wheat. More over, newly developed amiRNA technique has been showed specifically and efficiently in silencing single or multiple endogenous target genes in plant (Schwab et al., 2006). In the present study, we developed amiR-PDS against endogenous *PDS* in wheat by using Arabidopsis miR319 precursor as backbone (**Figures 1B, 7A**). As BSMV induced *PDS* silencing in wheat, overexpression of amiR-PDS in wheat seedlings resulted in a significant reduction in the mRNA levels of endogenous *PDS*, leading to leaf photobleaching (**Figure 7**). Therefore, the BSMV-based amiRNA overexpression system is very useful for functional characterizing endogenous protein-encoding genes in wheat.

Common wheat is one of the major food crops for the human diet, and its development is a very complicated event wherein the expansion and specialization of different cells are controlled by complex interactions of signaling and gene expression regulations. Yield and quality improvement of wheat largely depends on our knowledge of genes controlling wheat growth and development (Cakir et al., 2010). Therefore, understanding the involvement of miRNAs in plant development is crucial for wheat improvement, considering that miRNAs act as powerful endogenous regulators. The most widely applied reverse-genetic strategies to study the functions of certain miRNAs are overexpression and silence of the target miRNAs (Tang et al., 2010; Eamens et al., 2011), and these traditionally need tedious and time-consuming work to generate the stable transgenic plants (Yan et al., 2012). However, these techniques cannot be widely used in wheat due to its very low transformation efficiency which pose a technical challenge to produce numbers of wheat transformants. Here we elucidated BSMV-based miRNA overexpressing and miRNA silencing systems that can be used not only for studying the functions of endogenous miRNA genes but also for revealing the biological roles of the protein-coding genes by expression of amiRNAs against certain endogenous target genes.

In contrast, the BSMV-based miRNA overexpressing and miRNA silencing systems described here has some apparent advantages over the traditional functional assays for plant miRNAs. First, the BSMV-based miRNA overexpressing and silencing systems do not rely on stable transgenic wheat, and only need the simple BSMV infection technique for miRNA overexpressing or silencing. This is especially useful for functional identification of miRNAs whose knockout or knockdown might cause sporophytic or gametophytic lethality in transgenic lines. Second, the BSMV-based miRNA overexpressing and silencing systems are more efficient and quick, and miRNA overexpressing or silencing mediated phenotypes can be observed within 3 ∼ 4 weeks. Third, the BSMV-based miRNA overexpressing system using amiRNA and endogenous miRNA to target genes for silencing, it does not require to clone cDNA fragments of the target genes. It is much easy to obtain amiRNA clones, compared with the cDNA fragment used in BSMV induced gene silencing (Tang et al., 2010). The amiRNA precursor sequences can be easily obtained through overlapping PCR (Ossowski et al., 2008) or directly cloning of synthetic oligonucleotides into the stem region of pre-319 of Arabidopsis. This BSMV-based miRNA overexpressing and silencing systems will facilitate high-throughput functional characterization of endogenous miRNAs or the protein-encoding genes in wheat. As described above, we applied the systems to functionally characterize the target gene of miR156 via its transient down-regulation or up-regulation in wheat.

In summary, the BSMV-based miRNA overexpressing and miRNA silencing systems described in the present study have extraordinary potential not only for functional study of protein-encoding genes but also for functional characterization of miRNA genes in wheat.

## Author Contributions

HZ conceived and designed the experiments. CJ, RH, QC, SW, and MM conducted the experiments and analyzed the data. CJ and HZ wrote the manuscript. XL and MM read the draft of the manuscript.

## Conflict of Interest Statement

The authors declare that the research was conducted in the absence of any commercial or financial relationships that could be construed as a potential conflict of interest. The reviewer SH declared a shared affiliation, though no other collaboration, with the authors to the handling Editor, who ensured that the process nevertheless met the standards of a fair and objective review.

## Footnotes

1 http://www.mirbase.org

2 http://www.mirbase.org/cgi-bin/query.pl?terms=Triticum+aestivum+&submit=Search

3 http://www.mirbase.org/search.shtml

4 http://wmd3.weigelworld.org/cgi-bin/webapp.cgi

5 https://www.ncbi.nlm.nih.gov/nucest/CK196549
