# Supplementary material for: Virus-Based MicroRNA Silencing and Overexpressing in Common Wheat (Triticum aestivum L.)
Source: Front Plant Sci. 2017 Apr 10;8:500. doi: 10.3389/fpls.2017.00500 (PMC5385339; doi:10.3389/fpls.2017.00500)
Supplement: Supplementary file 1 [file Table_1.DOCX]

*Supplementary Material*

Virus-based microRNA silencing and overexpressing in common wheat (*Triticum aestivum* L.)

Chao Jian^1^, Ran Han^2^, Meng Ma^1^, Qing Chi^1^, Yanfeng Zhu^1^, Xiangli Liu^1^, Huixian Zhao^1,3,*^

*** Correspondence:** Corresponding Author: Huixian Zhao [hxzhao212@nwafu.edu.cn](mailto:hxzhao212@nwafu.edu.cn)

**Table S1 Primers used in this study**

| **Primer name** | **Sequences(5’→3’)** | **Purposes** |
| --- | --- | --- |
| P1  P2 | AACTGCCAATCGTGAGTAGGTT  CTCCTGTTCAGAACGTTTCAGAAGT | For *γb* RT-PCR |
| P3  P4 | ATATTAATTAACTGGATGAAAAAGCAGGGTGTTCC  TATGCGGCCGCCTACTTTCAGGAGGATTACCATCC | For cloning barley *PDS* fragment |
| P5  P6 | TGTCTTTAGCGTGCAAG  GATGATTTCGGTGTCACT | For endogenous *PDS* RT-PCR |
| P7  P8 | CGCTTCGGCTCCTCTCTCTCT  CGGACAAGATAGGGCTGAGGT | For cloning pre-miR156 |
| P9  P10 | CAAACACACGCTCGGACGCATA  GCGATGCCTTAAATAAAGATAAACCC | For cloning pre-amiRPDS |
| P11  P12 | GTTGTGTGGAATGTATGGAGC  GCTGTAATCACACTGGCTCA | For cloning STTM156/156 and STTM166/166 |
| P13 | GTCGTATCCAGTGCAGGGTCCGAGGTATTC  GCACTGGATACGACGTGCTC | For miR156 RT |
| P14 | GTCGTATCCAGTGCAGGGTCCGAGGTATTC  GCACTGGATACGACGGGGA | For miR166 RT |
| P15  P16 | CCTTCCGTGTTCCCACTGTTG  ATGCCCTTGAGGTTTCCCTC | For GADPH Real-time RT-PCR |
|  |  |  |
| P17 | TGACAGAAGAGAGTGAGCAC | For miR156 Real-time RT-PCR |
| P18 | TCGGACCAGGCTTCATTCCCC | For miR166 Real-time RT-PCR |
| P19 | GTGCAGGGTCCGAGGT | For miR156/166 Real-time RT-PCR |
| P20  P21 | GGGGACATCCGATAAAATTGG GGACCATTTCTCGATTTGTGC | For U6 Real-time RT-PCR |
| P22  P23 | GACCACCATGTTCGCCCACG  CCTTGTCTCCCAGGCTCACGT | For miR156 target gene Real-time PCR |
| P24  P25 | CAGTAAACTCATCGCCAAACAA  ACAAGCAAAGCAGGAGGGAC | For miR166 target gene Real-time RT-PCR |
| amiRPDS-I  amiRPDS-II  amiRPDS-III  amiPDS-IV | gaTAATCTGTTTAGAGGAATCAGtctctcttttgtattcc  gaCTGATTCCTCTAAACAGATTAtcaaagagaatcaatga  gaCTAATTCCTCTAATCAGATTTtcacaggtcgtgatatg  gaAAATCTGATTAGAGGAATTAGtctacatatatattcct | For cloning amiRPDS |
