# Supplementary material for: Virus-Based MicroRNA Silencing and Overexpressing in Common Wheat (Triticum aestivum L.)
Source: Front Plant Sci. 2017 Apr 10;8:500. doi: 10.3389/fpls.2017.00500 (PMC5385339; doi:10.3389/fpls.2017.00500)
Supplement: Supplementary file 2 [file Data_Sheet_1.PDF]

Virus-based microRNA silencing and overexpressing in common wheat (*Triticum aestivum* L.)

Chao Jian[1], Ran Han[2], Meng Ma[1], Qing Chi[1], Shijuan Wang[1], Yanfeng Zhu[1], Xiangli Liu[1], Huixian Zhao[1,3,*]

*** Correspondence:** Corresponding Author: Huixian Zhao hxzhao212@nwafu.edu.cn

1. Supplementary Method S1

Design of an artificial microRNA against *PDS* of wheat

A Web-based tool Web MicroRNA Designer (http://wmd3.weigelworld.org/cgi-bin/webapp.cgi) was used to design an amiRNA of wheat *PDS* gene (amiR-PDS). The detail steps are as follows:

Firstly, we cloned miR319 precusor (GenBank accession no. EU549293) pre-miR319, which was used as a backbone of amiR-PDS, from *Arabidopsis* by PCR method, and the PCR primers are listed in the following:

P9 （A） 5′-CAAACACACGCTCGGACGCATA-3′

P10（B） 5′-GCGATGCCTTAAATAAAGATAAACCC-3

Secondly, the resulting PCR product was inserted into clone vector PMD-19T to produce PMD-pre-319 to sequence. The correct clone was stored at -20℃ for using.

Thirdly, the coding sequenceof wheat *PDS* gene (GenBank accession no. FJ517553) and the pre-miR319 sequence were used to design an amiR-PDS using a Web-based tool Web MicroRNA Designer (http://wmd3.weigelworld.org/cgi-bin/webapp.cgi). The resulting mature amiR-PDS sequence is 5’-UAAUCUGUUUAGAGGAAUCAG-3’, and the primers for pre-amiR-PDS cloning are listed as follows:

I: 5′-gaTAATCTGTTTAGAGGAATCAGtctctcttttgtattcc-3′

II : 5′-gaCTGATTCCTCTAAACAGATTAtcaaagagaatcaatga-3′

III : 5′-gaCTAATTCCTCTAATCAGATTTtcacaggtcgtgatatg-3′

IV : 5′-gaAAATCTGATTAGAGGAATTAGtctacatatatattcct-3′

The primer locations are shown in the following Figure.

Finally, the development of pre-aimR-PDS by using PCR. PCR amplification steps, primer pairs and the template for each step are shown in the following Table. The volume of PCR reaction was 20 μl which contained:

Component	Amount
10XPCR buffer（with Mg2+）	5μl
dNTPs（2mM）	4μl
each primer（10μM）	2μl
template	2μl
Pfu taq	0.5μl
dH2O	34.5μl
total	50μl

The PCR reaction was performed at 95°C for 3min, followed by 35cycles of 95°C for 45s, 55~60°C for 30s, and 72°C for 4s, finally, 72°C for 10 min. And then, the PCR products of amplification step “4” (amiR-PDS precusor) was purified and inserted into BSMV vectors by T-A clone.

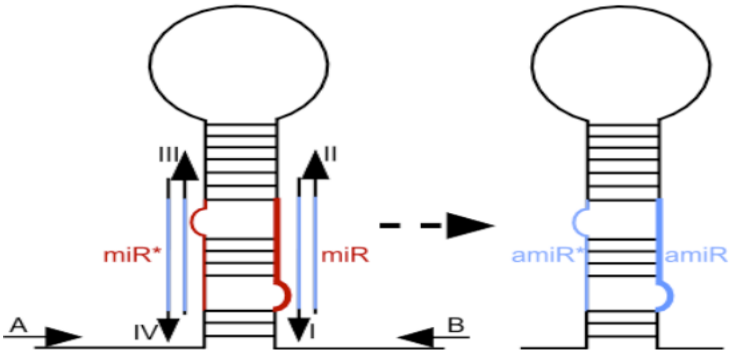

Figure The strategy of amiR-PDS cloning. The left shows the structure of pre-miR319 and the primer pairs used for cloning pre-amiR-PDS, The right presents pre-amiR-PDS.

Table Amplification step for amiR-PDS building process

Amplification step	Forward primer	Reverse primer	Template	PCR Product*
1	A	Ⅳ	PMD-pre-miR319	a
2	III	II	PMD-pre-miR319	b
3	I	B	PMD-pre-miR319	c
4	A	B	a+b+c	
